# Incidental learning in a multisensory environment across childhood

**DOI:** 10.1111/desc.12554

**Published:** 2017-04-26

**Authors:** Hannah J. Broadbent, Hayley White, Denis Mareschal, Natasha Z. Kirkham

**Affiliations:** ^1^ Centre for Brain and Cognitive Development Birkbeck, University of London London UK

## Abstract

Multisensory information has been shown to modulate attention in infants and facilitate learning in adults, by enhancing the amodal properties of a stimulus. However, it remains unclear whether this translates to learning in a multisensory environment across middle childhood, and particularly in the case of incidental learning. One hundred and eighty‐one children aged between 6 and 10 years participated in this study using a novel Multisensory Attention Learning Task (MALT). Participants were asked to respond to the presence of a target stimulus whilst ignoring distractors. Correct target selection resulted in the movement of the target exemplar to either the upper left or right screen quadrant, according to category membership. Category membership was defined either by visual‐only, auditory‐only or multisensory information. As early as 6 years of age, children demonstrated greater performance on the incidental categorization task following exposure to multisensory audiovisual cues compared to unisensory information. These findings provide important insight into the use of multisensory information in learning, and particularly on incidental category learning. Implications for the deployment of multisensory learning tasks within education across development will be discussed.

## RESEARCH HIGHLIGHTS

- Results indicate a reliable facilitatory effect of multisensory stimuli on learning between 6 and 10 years of age.
- Six‐year‐olds have a relative difficulty in using auditory‐only information for category learning.
- Multisensory integration may undergo a protracted developmental course through the early primary school years.
- The findings have implications for the deployment of multisensory learning tasks within primary education.

## 1 INTRODUCTION

Formal educational settings are vibrant multisensory environments. In order for an individual to make sense of the dynamic and cluttered environment, understanding that some cues signalled to more than one sense belong together, and being able to integrate information optimally from different sensory modalities whilst ignoring irrelevant information is imperative. Despite this, little is understood regarding age‐related changes in multisensory integration abilities, particularly within educational contexts, which would likely impact on our understanding of optimal learning conditions across development (Barutchu et al., 2011).

Environmental stimuli experienced through more than one sensory system can sometimes be considered advantageous when the pooling of redundant amodal cues is used to reduce perceptual uncertainty (Ernst & Banks, 2002). The use of mutually supportive multisensory information in a formal learning setting has intuitive appeal in that providing an individual with multiple cues should better support a representation. Indeed, numerous educational programmes have advocated the benefits of using multisensory information to facilitate learning, both in typically and atypically developing children (Bullock, Pierce, & McClelland, 1989; Luchow & Shepherd, 1981; Scott, 1993). There is, however, a paucity of systematic research examining the educational advantages of using multisensory stimulation within a learning setting (Mount & Cavet, 1995). This is particularly important considering the development of executive functions (e.g., inhibition, working memory) over the primary school years, which could impact on the effectiveness of using multisensory cues in a learning environment (Barutchu et al., 2011; Matusz et al., 2014). In addition, the protracted emergence of optimal integration of bimodal cues throughout childhood (Barutchu, Crewther, & Crewther, 2009; Gori, Del Viva, Sandini, & Burr, 2008) and developmental changes in sensory dominance on multisensory tasks (Nava & Pavani, 2013) also warrant an investigation into the extent to which children can benefit from multisensory information on basic learning tasks across development.

Research has shown that multisensory information can facilitate learning in adults (Fifer, Barutchu, Shivdasani, & Crewther, 2013; Lehmann & Murray, 2005; Seitz, Kim, & Shams, 2006; Shams & Seitz, 2008) and modulate attention in infants (Bahrick, Flom, & Lickliter, 2002; Bahrick & Lickliter, 2000; Gogate & Bahrick, 1998; Richardson & Kirkham, 2004). Moreover, both humans and non‐humans preferentially process intersensory redundant stimuli compared to unimodal information (for a review, see Baker & Jordan, 2015). The intersensory redundancy hypothesis (Bahrick & Lickliter, 2000; Bahrick, Lickliter, & Flom, 2004) posits that, in early development, the synchronous (temporal and spatial) presentation of information across two or more sensory modalities allows for enhanced detection and attention to properties of a stimulus. This suggests that amodal properties are less salient in unimodal presentation than when they are experienced redundantly across two senses. For instance, in a study with 5‐month‐olds, Bahrick and Lickliter (2000) found that infants are able to discriminate rhythm information when presented bimodally but not unimodally (visually or acoustically alone). The authors conclude that when amodal properties are presented unimodally, they do not recruit the same level of attention and are therefore not perceived or learned as effectively. In addition, Richardson and Kirkham (2004) found that 6‐month‐old infants were able to bind audiovisual events to locations. Following a familiarization phase to an audiovisual event, infants were able to remember (and update) the location of a visual event with just the provision of the audio information.

In sum, research on intersensory redundancy has provided insight into the use of cross‐modal matching and the coactivation (pooling) of redundant amodal stimuli in early development. There is, however, a big difference between using multisensory information to discriminate between stimuli or attend to events, and using multisensory information to enhance learning outcomes. Classroom‐based multisensory interventions typically focus on the use of different sensory equipment (i.e., using beads for counting, or using visual and kinaesthetic tools for teaching reading) in order to stimulate the learning experience (e.g., Alphabetic Phonics, Cox, 1985; or the Wilson Approach, Wilson, 1998). These techniques, however, do not necessarily acknowledge developmental changes in the ability to integrate multisensory information, and, importantly, there is little evidence to support their theoretical premises (Moats & Farrell, 1999). Thus, there is currently a large disparity between these two fields of knowledge, and whether the proposed benefits of simultaneously presenting stimuli to more than one sensory modality can be applied to basic learning tasks during the primary school years remains unclear.

To our knowledge, only a handful of studies have examined the effects of using multisensory information to support children's learning. For the most part, these have predominantly focused on reading or numerical remediation (Jordan & Baker, 2011; Jordan, Suanda, & Brannon, 2008; Thornton, Jones, & Toohey, 1983). For example, Joshi, Dahlgren, and Boulware‐Gooden (2002) reported improved reading skills in 6‐ to 7‐year‐olds, particularly in phonological awareness, decoding and comprehension, using multisensory teaching compared to a control group. Similarly, on a numerical learning task, Jordan and Baker (2011) found that preschool children aged 3 to 5 years were able to match numerosities more accurately with multisensory numerical stimuli than with unimodal. The authors attributed this finding to increased attention to the amodal property of number with the presentation of redundant information. Of note is that in each of these studies, participants were engaged in explicit, or intentional, learning tasks. Comparisons of explicit and incidental learning tasks, which differ on whether the participant is (respectively) instructed to learn or not, suggest there is little difference in the depth of processing and subsequent retention of information between these types of task in young children (Meulemans, Van der Linden, & Perruchet, 1998; Murphy & Brown, 1975). However, age‐related differences are observed in these different types of learning, with incidental learning abilities present earlier in development (Meulemans et al., 1998). Moreover, incidental learning may rely on neural systems that are distinct from those involved in explicit learning tasks (Gabay, Dick, Zevin, & Holt, 2015; Tricomi, Delgado, McCandliss, McClelland, & Fiez, 2006). It has not previously been examined whether multisensory information can facilitate learning in children on a task in which they are not overtly instructed to learn, and where the learning of information through unisensory or multisensory cues is incidental to the primary task. This is particularly important given that learning in naturalistic environments typically involves the processing of information presented to different senses and does not always arise from explicit instruction. For instance, on a mathematical learning task that involves counting different fruits and vegetables, the learning of concepts such as categorical information or other perceptual properties of the items may be incidental to the initial task, but are important comprehensively. Likewise, incidental learning of information relating to word architecture, narrative and syntactic structure may arise from being read aloud a story whilst looking at the words on the page.

For the most part, facilitative behavioural and cognitive advantages are found following the presentation of multisensory compared to unisensory cues. However, the senses do not interact in a homogenous way across development (Bremner, Lewkowicz, & Spence, 2012), and mature multisensory integration is not always observed until later in childhood. That is, the ability to reduce uncertainty on perceptual judgement tasks to adult levels by integrating information across sensory modalities has not been found until 8–12 years of age, depending on the task (Barutchu et al., 2009; Burr & Gori, 2012; Gori et al., 2008; Gori, Giuliana, Sandini, & Burr, 2012; Nardini, Bedford, & Mareschal, 2010; Nardini, Jones, Bedford, & Braddick, 2008; Petrini, Remark, Smith, & Nardini, 2014). For example, Nardini, Bales, and Mareschal (2015) found that although children as young as 4 years of age were faster and less variable in speeded responses to spatial location judgements using audiovisual compared to unimodal stimuli, pooling of the bimodal information was less efficient compared to that of older children and adults. As further support of a protracted emergence of multisensory integration, Hillock, Powers, and Wallace (2011) found that the audiovisual multisensory temporal binding window is still immature at 10–11 years. This was demonstrated by increasing the temporality of auditory and visual information, resulting in reduced fusion of the two modalities until this age.

The question therefore remains as to whether the use of multisensory information would facilitate learning to the same extent across development, particularly with consideration of educationally relevant stimuli that are complementary, although not redundant, and on a basic incidental learning task. For this reason, the current study was designed to examine the role of multisensory information on incidental category learning during an attentional vigilance task in children aged 6 to 10 years. It was hypothesized that there would be age‐related improvements in category learning, and an effect of sensory condition on incidental category learning across all groups. In light of research suggesting that mature integration of multisensory information is not seen until around 8 years of age, it was also hypothesized that there would be differential impacts of unisensory and multisensory cues on incidental category learning between 6 and 10 years of age.

## 2 METHODS

### 2.1 Participants

Data from 181 children were included in the study. Participants were selected from three separate school years (1, 3 and 5), resulting in three age groups; ‘6‐year‐olds’, *N *=* *60, mean age (years) = 6.05, *SD *= .52, (N = 25 males); ‘8‐year‐olds’, *N *=* *60, mean age = 8.26, *SD *= .31 (*N *=* *25 males); and ‘10‐year‐olds’, *N *=* *61, mean age = 10.20, *SD* = .41 (*N *=* *32 males). Participants in each age group were randomly allocated to one of three learning conditions, in a between‐subjects design (*N *=* *20 per condition, except *N *=* *21 in 10‐year‐olds for Audiovisual condition); Visual (unisensory), Auditory (unisensory) or Audiovisual (multisensory).

Children were recruited from local primary schools and informed written parental consent was obtained for each participant, in accordance with the university ethics committee guidelines. All participants had normal hearing and normal (or corrected‐to‐normal) vision, and no known developmental or neurological disorder, as assessed on the parental consent form. All testing was conducted in a quiet room within the participant's school and children were rewarded for participating with a certificate and stickers. Testing sessions for each participant lasted approximately 20 minutes.

### 2.2 Stimuli

The Multisensory Attention Learning Task (MALT) is a novel computerized category‐learning task, based on a modified version of a classic continuous performance task, and adapted for use with primary school aged children. The MALT was developed to examine the role of unimodal and multimodal information on attentional vigilance and incidental learning of categorical information. Visual stimuli consisted of seven different animal line drawings, subtending a 3° visual angle, and presented on a 15ʺ laptop screen approximately 50 cm in front of the participant. Animal stimuli consisted of one target animal (‘frog’) and six non‐target animals (‘owl’, ‘dog’, ‘goat’, ‘pig’, ‘elephant’, and ‘cat’). All visual images were forward facing depicting a head and body with (front) legs for consistency and to maintain a level of similarity across stimuli. Auditory stimuli consisted of congruent animal sounds, consistent with the different visual animal stimuli. Auditory stimuli were presented at 44 kHz and around 70–75 dB through closed‐back headphones. Stimuli were presented using the Psychophysics Toolbox extension for MATLAB (Brainard, 1997).

**Figure 1:**
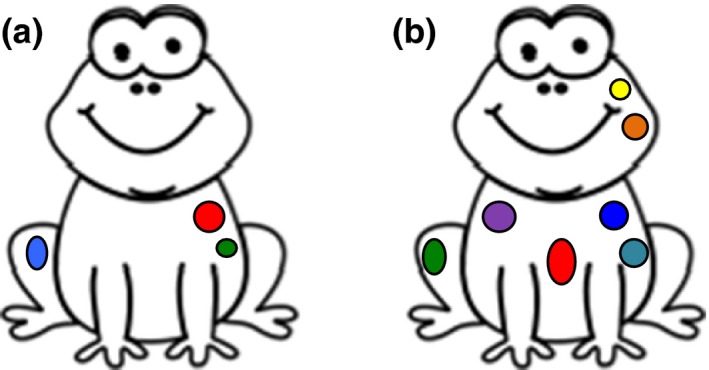
Exemplars of target stimuli from visual categories 1 and 2 (A and B, respectively)

In the unimodal visual learning condition, contrasting visual features were used to distinguish between two different categories (‘families’) of frogs. Frogs from family 1 had few spots (2 or 3), varying in size and colours across category members (10 within‐category members). Members within family 2 had many spots (7 or 8), varying in colours and size consistent with members from family 1 (10 within‐category members). For exemplars of targets from the two visual categories, see Figure 1. Non‐target animals were similarly marked with spots of varying colours, size and number, for consistency across stimuli. In the visual learning condition, auditory stimuli remained consistent across exemplars. That is, for target stimuli (frogs), only one of the two auditory‐cue ‘families’ (see below for further details) was used, counterbalanced across participants.

In the auditory learning condition, only unimodal auditory features were used to differentiate family members. Auditory stimuli were presented for 300 ms, consistent with visual presentation times. The visual ‘family’ for target stimuli remained consistent and was counterbalanced across participants. Target stimuli ‘families’ were distinguishable by two different frog croaks, each with a double‐croak (‘*rib‐bit*’) sound. Family 1 exemplars croaked with a ‘high and long‐short’ sound, whilst family 2 exemplars croaked with a ‘deep and short‐long’ croak (manipulated using ‘Audacity Digital Audio Editor Software’). Five different pitches of croak were used as a variant to denote different within‐family members, varying in 0.5 semitone intervals. All other sound file properties remained consistent across and within families.

In the multimodal audiovisual learning condition, both visual and auditory features could be used to discriminate category membership. For example, family 1 members had few spots (visual) and a long‐short croak (auditory), whilst family 2 members had many spots and a short‐long croak. The two possible combinations of categorizing audiovisual features were counterbalanced across participants.

#### 2.2.1 Stimuli discrimination

An initial pilot study with 17 participants aged between 6 and 10 years showed that exemplars from the two different families were equally discriminable for both visual and auditory conditions. Participants were presented with 16 pairs; six ‘identical’ and six ‘different’ (between‐family) pairs, as well as four ‘different but within‐family’ pairs, and asked whether they were the ‘same or different’. This was done for both the visual and auditory learning condition stimuli (counterbalanced order across participants), resulting in a total of 32 discrimination trials for each participant. All participants were able to successfully complete the task and no reliable difference between visual and auditory discrimination scores were found, t(16) = −1.16, *p *=* *.261.

### 2.3 Procedure

As a measure of auditory working memory, each participant initially completed the Digit Span Backwards (DSB) task from the British Ability Scales–II (BAS‐II; Elliott, Smith, & McCulloch, 1996). Before presentation of the MALT, a short audio and visual detection task was conducted in which participants were familiarized with the task stimuli. Participants were shown one of each animal in turn and asked whether they were able to hear and see the exemplar. Only participants who answered affirmatively for each of the seven exemplars continued with the task.

#### 2.3.1 Multisensory attention learning task (MALT)

**Figure 2:**
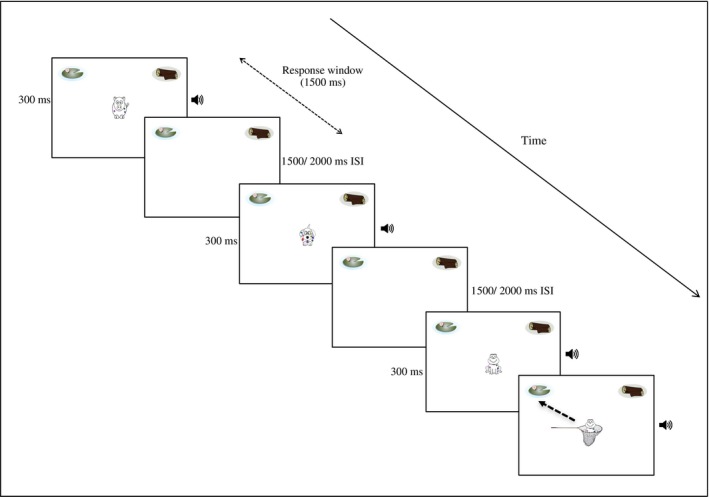
MALT presentation order. The final depicted screen would appear following a correct key‐press response to the target stimulus, with dashed arrow indicating direction of movement to correct category habitat

For the computerized MALT task, participants sat approximately 50 cm in front of a 15ʺ laptop screen. Participants were instructed to press the space bar as quickly as possible whenever a frog (target animal) appeared on the screen, whilst inhibiting a response to any other animal stimuli. Participants were told to rest their hand over the response bar to be ready for each trial. The task screen consisted of a white screen with an image of a lily pad in the top left‐hand corner and an image of a log in the top right‐hand corner. On each trial, an animal image appeared individually in the centre of the screen for 300 ms. If the space bar was (correctly) pressed after the presentation of a target stimulus, the same frog reappeared in a ‘net’ (see final slide on Figure 2). The frog then immediately travelled to the top left‐ or top right‐hand corner of the screen to the correct frog habitat (i.e., unbeknown to the participants, frog exemplars from one family consistently travelled to the lily pad habitat, whilst frog exemplars from the other family travelled to the log habitat, counterbalanced across participants). Travel time to habitat lasted 2000 ms. The corresponding audio file for that frog was also played simultaneously and three times until the frog reached the correct habitat. This was for consistency with exposure to the visual stimuli for incidental learning of categorical information.

Following movement to the habitat, the target image was then paused for an additional 1000 ms to avoid disorientation caused by an immediate appearance of the next stimulus. If the button had been pressed incorrectly for a non‐target animal, no feedback was given and the task continued to the next trial after either a 1500 ms or a 2000 ms inter‐stimulus interval (ISI). These ISIs were selected in line with research that shows these timings to be optimal for task performance when used with children (e.g., Chee, Logan, Schachar, Lindsay, & Wachsmuth, 1989; Okazaki et al., 2004). For a schematic of the MALT presentation sequence, see Figure 2.

The task consisted of 200 trials, separated into four blocks by a motivation screen to allow for rest‐breaks. Across the task, target stimuli (frogs) were presented on 40% of trials (80 trials; 40 exemplars from each family). Twenty of each non‐target (distractor) stimuli were presented randomly throughout the task. Completion of the task was determined either by 50 correct responses to frog targets (calculated cumulatively across trials from task beginning), or until the maximum 200 trials were completed. Participants were therefore scored as having reached criterion or not. Data were analysed only from those who met the 50‐correct target responses criteria. As such, all participants included in the analyses had received the same number of category learning trials (having observed 50 frogs travelling to their correct habitat). Only one 6‐year‐old participant and one 8‐year‐old did not meet criterion.

#### 2.3.2 Category identification test

To examine the extent of incidental category learning on the MALT, participants were subsequently asked to complete a category identification task. Participants were not made aware that they would be tested on category knowledge before this point, nor had they been informed that they should try and learn any aspect from the task before the initiation of the study. Eight exemplars from each category (of the given learning condition) were presented in a random order. Participants responded to whether the frog had lived at the lily pad or the log during the game. An initial pilot study with 16 participants aged 6 to 10 years found an increased occurrence of alternate responses being made (lily pad, log, lily pad, log, etc.) when asked to respond using the keyboard. Participants were therefore asked to respond verbally and the researcher would press the correct habitat image positioned on the keyboard on keys ‘z’ and ‘m’, respectively. Participants viewed each frog individually, and no feedback was given throughout the identification task. Total correct categorization responses were recorded. Following the categorization test, as a measure of explicit categorization knowledge, participants were then asked, ‘Can you tell me how you decided where each frog lived? What made them belong to each family?’

## 3 RESULTS

### 3.1 Auditory working memory

Digit Span Backwards (DSB) raw ability scores were converted to standardized *T*‐Scores and compared across groups using a one‐way analysis of variance (ANOVA). No significant difference was found between groups; 6‐years: Mean (*SD*) = 56.60 (9.89); 8‐years = 54.03 (9.37); 10‐years = 55.07 (9.92), (*F*(2, 180) = 1.07, *p *=* *.345), showing that participants in each group were performing at a cognitive level expected for their age.

### 3.2 Multisensory Attention Learning Task (MALT)

To examine performance across groups on aspects of sustained attention on the learning element of the MALT, trials to criterion and number of errors were calculated.

#### 3.2.1 Trials to criterion

The mean number of learning trials on the MALT in order to reach the criterion of 50 correct target responses was calculated for each group. Results of a univariate ANOVA with two between‐subjects factors of Age Group (3 levels: 6, 8, and 10) and Condition (3 levels: V, A, and AV) found a significant main effect of Age Group, *F*(2, 172) = 4.44, *p *=* *.013, partial η^2^ = .05, but not of Condition (*F *<* *1), with 6‐year‐olds requiring a significantly greater number of trials (Mean = 146.98, *SD* = 8.05) to reach criterion than 8‐year‐olds (Mean = 143.18, *SD* = 7.92), *p *=* *.025, and trend for more trials than 10‐year‐olds (Mean = 143.67, *SD* = 6.73), *p *=* *.055. No differences were seen between 8‐ and 10‐year‐olds (*p *>* *.05).

#### 3.2.2 Errors on MALT

A univariate ANOVA to analyse mean number of commission errors (i.e., incorrectly responding to a non‐target item) across Age groups and Conditions (see Table 1) found a significant main effect of Age Group, *F*(2, 172) = 5.05, *p *=* *.007, partial η^2^ = .06, but not Condition (*F *<* *1), driven by 6‐year‐olds making significantly more commission errors than 10‐year‐olds, *p *=* *.009 (Bonferroni‐corrected pairwise comparisons).

**Table 1:** Mean number of commission and omission errors on MALT for each condition across groups

		Mean number of errors (*SD*)		
		6 years	8 years	10 years
Commission errors	V	13.25 (13.95)	9.00 (8.65)	9.45 (13.20)
	A	16.50 (12.75)	8.60 (6.57)	8.90 (8.79)
	AV	13.70 (10.64)	11.80 (12.60)	7.29 (7.52)
Omission errors	V	5.75 (4.20)	3.55 (4.22)	3.15 (4.21)
	A	5.80 (4.91)	1.30 (1.81)	4.20 (4.49)
	AV	3.75 (2.79)	3.90 (6.16)	2.62 (3.53)

Mean number of omission errors (i.e., failing to respond to the correct target) across Age groups and Conditions (Table 1), analysed as above, found a significant main effect of Age, *F*(2, 172) = 4.59, *p *=* *.011, partial η^2^ = .05, but not Condition (*F *<* *1). Pairwise comparisons (Bonferroni‐corrected) found 6‐year‐olds made significantly more omission errors than 8‐year‐olds (*p *=* *.015) and there was a trend for 6‐year‐olds to make more errors than 10‐year‐olds (*p *=* *.061).

#### 3.2.3 Category identification test

**Figure 3:**
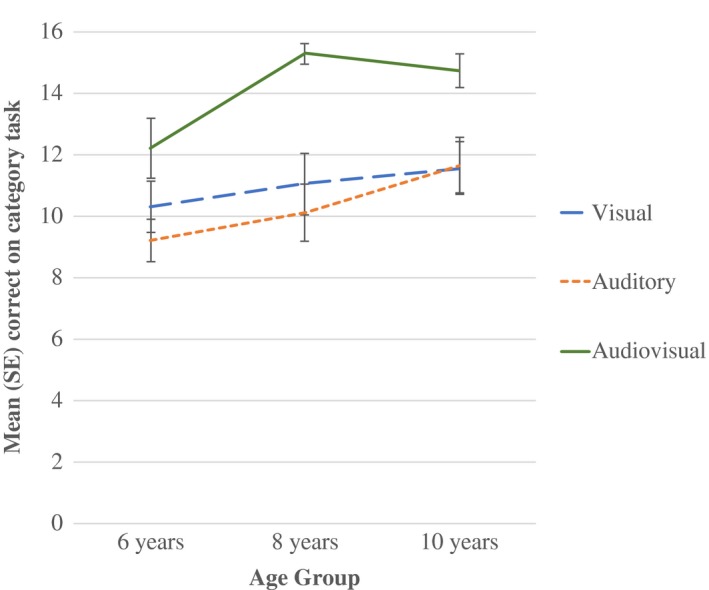
Mean (*SE*) correct on category identification test in each age group across learning conditions

As a measure of incidental category learning, mean number correct on the category identification task was calculated for each age group and compared across learning condition (Figure 3). Results of a univariate ANOVA with two between‐subjects factors of Age Group (3 levels: 6, 8, and 10) and Condition (3 levels: V, A, and AV) found no significant Age Group by Condition interaction (*F *<* *1). However, significant main effects of Age Group, *F*(2, 168) = 5.23, *p *=* *.006, partial η^2^ = .06, and Condition, *F*(2, 168) = 17.42, *p *<* *.001, partial η^2^ = .17, were identified. Pairwise comparisons (Bonferroni‐corrected) for Age Group found that 6‐year‐olds performed reliably below 10‐year‐olds (*p *=* *.007), with no differences between 6 and 8 years, or 8 and 10 years (*p *>* *.05, for all). For Condition, pairwise comparisons indicated that participants scored significantly higher following the Audiovisual learning condition (Mean = 14.07) than either the Auditory (Mean = 10.32) or Visual‐only (Mean = 10.97) conditions (*p *<* *.001 for both). No difference was found between Auditory and Visual groups (*p *=* *.996).

To examine whether incidental categorization performance differed from chance, data were analysed for each Age group and Condition using one‐sample *t*‐tests with a test value of 8. Six‐year‐olds were found to score significantly above chance on the Visual‐only (*t*(19) = 2.73, *p *=* *.013) and Audiovisual (*t*(19) = 4.23, *p *<* *.001) conditions, but not in the Auditory‐only condition (*p *=* *.095). The 8‐ and 10‐year‐olds scored significantly above chance on all learning conditions (*p *>* *.05, for all), indicative of a high level of categorization performance in these groups across conditions.

**Figure 4:**
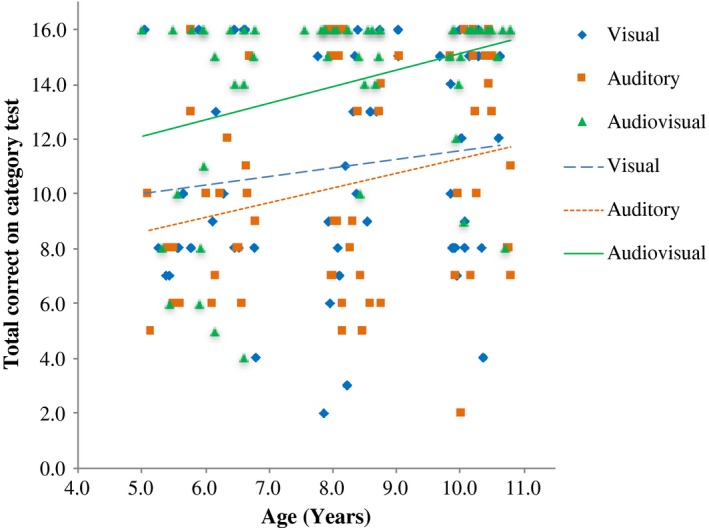
Relationship between chronological age and number correct on category identification task in each learning condition

An examination of the relationship (Pearson's *r*) between age (collapsed across groups) and performance on the category identification task for each condition indicated a significant positive correlation in the Audiovisual learning condition, *r *=* *.334, *p *=* *.011, and a trend for a positive correlation in the Auditory‐only learning condition, *r *=* *.249, *p *=* *.055, but not in the Visual‐only learning condition (*p *=* *.319). Data are presented in Figure 4.

An investigation of the relationships (Pearson's *r*) between incidental learning (total correct on category task) and auditory working memory (DSB), sustained attention skills (omission errors) and inhibitory control skills (commission errors) found no significant correlations across any age groups or conditions (*p *>* *.05, for all).

#### 3.2.4 Explicit categorization knowledge test

**Figure 5:**
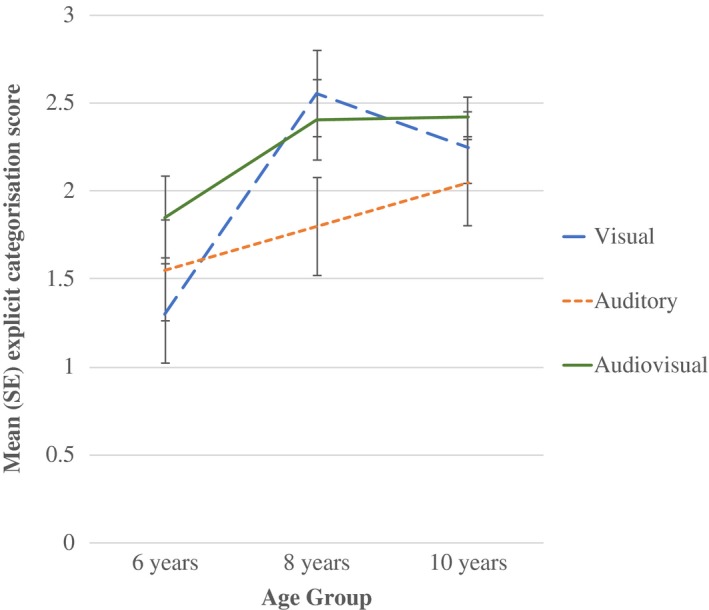
Mean (*SE*) explicit category knowledge score for each age group across learning conditions

As well as an examination of incidental knowledge, following the category identification task, each participant was asked to state verbally what they judged the differences between the two families of frogs to be and how they reached their categorization choices. Verbal responses were scored as follows; don't know/none given = 0 points, related categorical description given but inaccurate (e.g., ‘they had different coloured spots’) = 1 point, partially correct family description (i.e. citing 1 feature but not both in AV condition, e.g., number of spots, but no mention of auditory features) = 2 points, fully correct family description (i.e. ‘different number of spots and different croak sounds’ in AV condition or ‘croaks to log were deeper than croaks to lily pad’ for A condition) = 3 points. A mean explicit categorization score was calculated for each group and condition (Figure 5). Although a high correlation was found between incidental and explicit scores (*r *=* *.455, *p *<* *.001), results of a univariate ANOVA with two between‐subjects factors of Age and Condition for explicit knowledge data indicate a different pattern of performance than seen in the incidental knowledge test. That is, although results found a main effect of Age Group, *F*(2, 172) = 7.86, *p *=* *.001, partial η^2^ = .08, with 6‐year‐olds significantly less able to express the correct reason for categorizing than the older two groups (*p *=* *.002 and *p *=* *.003), no main effect of Condition, *F*(2, 172) = 2.22, *p *=* *.112, partial η^2^ = .03, was found. This suggests that there is an age‐related difference in the ability to verbally express categorization knowledge compared to the incidental learning element of the task.

#### 3.2.5 Discrimination task

To examine the saliency and discriminability level of the visual and auditory features of target exemplars, the same discrimination task as used in the initial pilot study (see above description in Stimuli discrimination) was conducted with 15 participants randomly selected from each age group (including five participants from each condition). Mean accuracy score for visual and auditory discriminators was calculated for each age group. Results of a one‐way ANOVA found a significant difference across groups between visual and auditory score; *F*(2, 42) = 4.17, *p *=* *.023, driven by 6‐year‐olds scoring significantly below 10‐year‐olds in visual discrimination. Paired samples *t*‐tests to examine differences in visual and auditory accuracy scores for each age group separately revealed significantly lower visual than auditory discrimination ability only in 6‐year‐olds; Mean (*SD*) visual = 11.33 (2.35), auditory = 12.47 (1.46), *t*(14) = −2.20, *p *=* *.045. No significant difference between visual and auditory discrimination ability was found for 8‐year‐olds; Mean (*SD*) visual = 13.07 (1.39), auditory = 13.27 (.88), *p *=* *.647, or for 10‐year‐olds; Mean (*SD*) visual = 13.33 (2.02), auditory = 12.80 (2.51), *p *=* *.217.

## 4 DISCUSSION

The current study used a novel category‐learning task to examine the effects of unisensory and multisensory cues on incidental category learning across middle childhood. As expected, the results indicate a significant improvement in incidental learning from 6 to 10 years of age. In addition, as early as 6 years of age in this study, children demonstrated greater performance on an incidental categorization task following exposure to multisensory (audiovisual) cues compared to unisensory information (visual or auditory alone).

Multisensory information has previously been shown to improve encoding (Bahrick & Lickliter, 2012) and better facilitate subsequent learning compared to unisensory stimulation in children as young as 3 to 4 years of age (Jordan & Baker, 2011). Similarly, on speeded RT tasks, children as young as 4 years of age were able to integrate audiovisual information to improve performance to a greater extent than with the presentation of unimodal stimuli, but were less efficient than older children and adults (Nardini et al., 2015). Other developmental studies that have examined multisensory integration on tasks that did not require speeded responses also report the pooling of bimodal signals to be sub‐optimal until even later in childhood, around 8 to 12 years of age (Gori et al., 2008; Gori et al., 2012; Nardini et al., 2010; Nardini et al., 2008; Petrini et al., 2014). In sum, such findings suggest that although multisensory information may be pooled to a certain extent at this young age, mature integration of bimodal signals undergoes a more protracted developmental course.

The emphasis in the current study was on incidental category learning during a sustained attention task. This differed from the aforementioned previous studies and their focus on developmental changes in the pooling of redundant cues on explicit learning or perceptual tasks. Incidental acquisition of information occurs across multiple learning tasks in educational environments (Postman, 1964), and is therefore an important area of focus for research examining the role of multisensory stimuli on learning. In the current study, the simultaneous presentation of complementary visual and auditory information, in which both features were informative to family membership, resulted in enhanced performance on the incidental learning of categories across all age groups.

Although no significant interaction between age and learning condition was found, others have found that the pooling of multisensory cues may become more advanced with age (Barutchu et al., 2009; Gori et al., 2008; Gori et al., 2012). The emphasis on learning in the current study may therefore underlie the differences in findings from studies examining the development of pooling bimodal cues. That said, despite a lack of reliable difference in the pattern of performance with age in the current study, some age‐related changes in the benefits of multisensory cues were identified. For instance, performance on the category identification task following audiovisual learning positively correlated with age, and with a trend for a positive relationship between age and auditory‐only learning. In contrast, performance following visual‐only learning did not correlate with age. These results are therefore somewhat in line with previous findings that argue for a refining of the ability to use multisensory information across this age span (e.g., Nardini et al., 2015). This would afford the conclusion that the use of multisensory cues for learning may still undergo some development during the primary school years. Of note, however, is that there was also a trend for improved performance with age in the auditory‐only condition, suggesting that these findings may reflect age‐related changes in the use of auditory information to support learning. This is particularly supported by our findings that 6‐year‐olds performed at chance following learning with auditory‐only cues, but above chance with visual and audiovisual cues. Others have also reported age‐related improvements in auditory processing throughout childhood and into adolescence that may affect responses to perceptual training (Huyck & Wright, 2013). Similarly, differences in the processing of visual and auditory stimuli with age have been seen on multisensory tasks, with children and adolescents, compared to adults, showing reduced processing of auditory distractors compared to visual and bimodal (Downing, Barutchu, & Crewther, 2014).

In this study, therefore, although younger children used visual information (both in the visual‐only and multisensory conditions) to the same level as older children, changes with age were seen in the extent to which auditory cues were considered useful for learning. Initially, this could be considered a matter of cue saliency, with the auditory stimuli not having been as salient as the visual information. However, this explanation is contested by our seemingly contradictory findings that children at this age were less able to discriminate between visual targets than between auditory exemplars, but with an equal level of discriminability between the different modality exemplars above 8 years of age. Furthermore, no differences in categorical learning were found between unisensory visual and auditory cues in any group in this study, including 6‐year‐olds, suggesting that visual and auditory stimuli were equally salient and usable.

As an alternative explanation, the findings may allude to a visual processing bias in younger children. This is in contrast to findings of an auditory processing dominance in young children, with a change to visual dominance in older children and adults (Napolitano & Sloutsky, 2004; Sloutsky & Napolitano, 2003). By 4 years of age there is some flexibility observed in terms of modality dominance that is dependent on the task demands, wherein stimuli are only processed in the preferred modality when different sensory cues are of equal salience (Robinson & Sloutsky, 2004). Therefore, children aged 6 years may already demonstrate visual dominance on tasks such as the one presented here. Given that no age and condition interactions were identified, however, such conclusions can only be met tentatively. Indeed, it is also worth noting that neither of the oldest two groups demonstrated this visual processing dominance, despite robust findings of visual modality dominance in older children and adults on other tasks (Koppen & Spence, 2007; Sinnett, Soto‐Faraco, & Spence, 2008; Spence, 2009).

As well as an analysis of group differences on an incidental category‐learning task, we also reported the findings from the attention trials on the main MALT task. Here, no differences were found across the different MALT learning conditions, suggesting that effects of condition in incidental learning were not related to the attentional aspects of the original task. Although differences were seen between age groups, all groups demonstrated a comparable pattern of performance.

Furthermore, although 6‐year‐olds required more trials to criterion, all participants included in the analyses experienced a total of 50 target exemplars travelling to the two habitats before the category task was presented. Analyses of these learning task parameters therefore only highlight age group differences rather than differences across learning conditions. This is in line with what would be expected on measures of sustained attention in these age groups. As such, age‐related differences on this aspect of the task likely reflect improvements in speed of processing visual and auditory information, developmental changes in levels of inhibition (Levy, 1980), as indicated in a reduction in commission errors, and improved attention, as measured by decreasing omission errors, from the youngest to oldest age groups.

As well as a measure of incidental category learning, the current study examined explicit categorical knowledge across groups. A difference was found in the pattern of performance in the incidental learning compared to the explicit knowledge tests, with no effect of condition observed in the latter, and the youngest children (6‐year‐olds) demonstrating particular difficulty in expressing correct categorical information. While no feedback was given on the incidental categorization task, this finding may be related to the participants being made aware of categorical differences both in the incidental task and being posed a question of this nature in the subsequent explicit knowledge task. This may have cued participants to devise a plausible explanation for categorical differences. Thus, being asked to verbally express categorical information before the presentation of the incidental category identification task may have resulted in a levelling of performance across the two different tests. Alternatively, this finding may be reflective of different processing systems for explicit and incidental learning (Gabay et al., 2015; Tricomi et al., 2006).

Our results raise the question as to whether similar findings would also be observed not only on other novel categorical learning tasks, but also other learning tasks such as associative learning, and in different domains such as language and numerical learning. Jordan and Baker (2011) found that in young children aged 3 to 5 years, learning to match numerosities was facilitated when given multisensory rather than unisensory information about the number. A key difference in these studies is in the nature of incidental learning in the current task as opposed to explicit mathematical concept learning in the above‐mentioned study. A further difference is that our analyses were not concerned with speed of responses, but rather the accuracy of categorical selection. In addition, in the study by Jordan and Baker (2011), audiovisual trials provided a greater total amount of stimulation in comparison to unimodal trials. That is, only on audiovisual trials were participants exposed to both visual and auditory information. This may have resulted in enhanced arousal to stimulus properties and subsequent representations. In the current study, all learning trials (regardless of learning condition) included both auditory and visual events, with learning conditions differing only on the basis of the informative nature of the cues (i.e., the features that could be used for categorical judgements). Findings from the current study therefore refute the assumption that better performance in a multisensory learning condition compared to unisensory is a result of enhanced stimulation from multisensory trials. In conclusion, even in light of the differences in tasks used across studies, the comparable results of improved learning following exposure to multisensory cues compared to unisensory, even in children as young as 6 years, is a robust finding.

As mentioned previously, on some tasks, multisensory integration is not as efficient in young children as it is in older children and adults (Burr & Gori, 2012; Gori et al., 2008; Nardini et al., 2010; Nardini et al., 2008), a finding somewhat reflected in the current study. Conclusions from earlier studies imply that combining audio and visual stimuli either at the level of attention or at a neural level of stimuli integration may be more difficult for younger children and therefore not facilitate learning to the same extent as in older children. However, there are likely to be numerous cortical and subcortical mechanisms involved in multisensory integration that may develop at different rates (e.g., Molholm et al., 2002; Noesselt et al., 2007; Stekelenburg & Vroomen, 2007). This may underlie the disparity in the reported ages at which mature levels of multisensory facilitation are observed, particularly given that performance on different multisensory tasks may be associated with distinct neural substrates. The examination of multisensory cues on incidental category learning in children younger than 6 years of age would be an important avenue for future research in order to elucidate this further.

In the current study, it was only the nature of cues for categorical learning that differed across learning conditions. It is not clear therefore whether multisensory stimulation in some learning contexts would have a distracting effect on performance or would lead to increased focus of attention; particularly when multimodal stimuli are not task‐related, as would typically be encountered within a learning environment. For instance, difficulties in encoding unisensory cues have been found when multisensory properties compete for attention (Lickliter & Bahrick, 2004). Given known developmental changes in attention, there may also be differing patterns of response to multisensory distraction across development. Further research should therefore examine the use of unimodal and bimodal noise (distractors), or an increased working memory load within and between modalities on a similar learning task.

This study provides important insight into the use of multisensory information in an educational environment on incidental category learning. The intersensory redundancy hypothesis (IRH; Bahrick & Lickliter, 2012) posits that the pooling of multisensory cues presented in synchrony leads to enhanced perception. Given the nature of the current task, theoretical assumptions of the IRH can only go some way to explaining the current results of enhanced category learning following multisensory cue exposure compared to unisensory. Essentially, the current study included complementary but not redundant amodal stimuli in order to better emulate sensory information typically found in learning environments. Even in light of this difference, the results suggest a reliable facilitatory effect of multisensory stimuli presentation between 6 and 10 years of age. Moreover, our results are in accord with findings that multisensory integration (particularly with the integration of auditory and visual information) may undergo a protracted developmental course through the early primary school years. This has particular implications for the deployment of multisensory learning tasks within primary education. In particular, multisensory information may not be as beneficial to younger children when information from a single sense is dominant. For instance, the results are indicative of a relative difficulty in the use of auditory information to support category learning in 6‐year‐olds, unless combined with complementary visual information. This has implications for the use of auditory information on categorical learning tasks in children below 8 years of age. Where the simultaneous presentation of auditory information with visual cues may better support a representation and subsequent learning, this may be particularly relevant for younger children who demonstrate poorer performance than older children on unimodal auditory tasks.
